# Liquid Biopsies in Hepatocellular Carcinoma: Are We Winning?

**DOI:** 10.3390/jcm9051541

**Published:** 2020-05-20

**Authors:** Tudor Mocan, André L. Simão, Rui E. Castro, Cecília M. P. Rodrigues, Artur Słomka, Bingduo Wang, Christian Strassburg, Aliona Wöhler, Arnulf G. Willms, Miroslaw Kornek

**Affiliations:** 1 Octavian Fodor Institute for Gastroenterology and Hepatology, Iuliu Haţieganu, University of Medicine and Pharmacy, 400162 Cluj-Napoca, Romania; mocan_tudor@yahoo.com; 2 Research Institute for Medicines (iMed.ULisboa), Faculty of Pharmacy, Universidade de Lisboa, 1649-003 Lisbon, Portugal; Adlsimao@ff.ulisboa.pt (A.L.S.); ruieduardocastro@ff.ulisboa.pt (R.E.C.); cmprodrigues@ff.ulisboa.pt (C.M.P.R.); 3 Department of Pathophysiology, Nicolaus Copernicus University in Toruń, Ludwik Rydygier Collegium Medicum, 85-094 Bydgoszcz, Poland; artur.slomka@cm.umk.pl; 4 Department of Internal Medicine I, University Hospital of the Rheinische Friedrich-Wilhelms-University, 53127 Bonn, Germany; bingduo.wang@ukbonn.de (B.W.); Christian.Strassburg@ukbonn.de (C.S.); 5 Department of General, Visceral and Thoracic Surgery, German Armed Forces Central Hospital Koblenz, 56072 Koblenz, Germany; alionawoehler@bundeswehr.org (A.W.); arnulfwillms@bundeswehr.org (A.G.W.)

**Keywords:** biomarker, circulating tumor nucleic acids, circulating tumor cells, extracellular vesicles, proteomics and metabolomics

## Abstract

Hepatocellular carcinoma (HCC) represents the sixth most common cancer worldwide and the third most common cause of cancer-related death. One of the major problems faced by researchers and clinicians in this area is the lack of reliable disease biomarkers, which would allow for an earlier diagnosis, follow-up or prediction of treatment response, among others. In this regard, the “HCC circulome”, defined as the pool of circulating molecules in the bloodstream derived from the primary tumor, represents an appealing target, the so called liquid biopsy. Such molecules encompass circulating tumor proteins, circulating tumor cells (CTCs), extracellular vesicles (EVs), tumor-educated platelets (TEPs), and circulating tumor nucleic acids, namely circulating tumor DNA (ctDNA) and circulating tumor RNA (ctRNA). In this article, we summarize recent findings highlighting the promising role of liquid biopsies as novel potential biomarkers in HCC, emphasizing on its clinical performance.

## 1. Introduction

Between 1990 and 2015, the incidence of hepatocellular carcinoma (HCC) increased by 75%, with this type of cancer being accountable roughly for more than 810,000 deaths in 2015 [1,2]. At around the same time, the Italian Liver Cancer Group showed that the 5-year overall survival rate in HCC was 32.7% in semi-annually surveilled patients, 25.2% in annually surveilled patients, and 12.2% in symptomatic patients [3]. These results strongly suggest that a semi-annual HCC screening might prolong lifetime as a result of early HCC monitoring/surveillance [3]. Hence, the European Association for the Study of the Liver (EASL) and other liver cancer interest groups emphasized the urgent need of suitable hepatic cancer biomarkers. In fact, while HCC management has changed dramatically in past recent years, there has not been much progress in the discovery of reliable and predictive biomarkers obtained through liquid biopsies in HCC.

Alpha-fetoprotein (AFP) was one of the first biomarkers of diagnosis evaluated in the blood of HCC patients. Due to the promising results, the 2003 HCC guidelines from the British Society of Gastroenterology recommended both AFP and abdominal ultrasound for HCC surveillance [4]. Nonetheless, it has since been shown that AFP is relatively insensitive under certain circumstances, as it is only elevated in the blood in 40%–60% of HCC cases. Further, AFP levels can also be found elevated in non-HCC patients, including non-cancerous chronic liver diseases, intrahepatic cholangiocarcinoma and metastatic colon cancer [5]. Additional potential diagnostic biomarkers for HCC have also been studied in HCC, singularly or in combination, including des-γ-carboxyprothrombin (DCP), AFP lectin fraction (AFP-L3), and glypican 3, among others [6]. Unfortunately, available data has shown similar or only slightly better sensitivities and specificities of these biomarkers when compared to AFP, with none being used in clinical practice.

The indications for tissue biopsy in HCC have evolved over time [7]. The development of high-throughput technologies has allowed the detection of molecular drivers of tumorigenesis and of specific DNA mutations in tumor biopsy samples, which may be able to predict response or resistance to targeted therapies [8]. The same technologies have also highlighted the significance of morphological and genetic tumor heterogeneity when considering a liver biopsy for diagnostic or prognostic purposes. For instance, it was shown that somatic mutations generally occur early during tumorigenesis and tend to propagate in many clones, whereas later mutations exist only in some clones (spatial heterogeneity) [9]. Additionally, treatment initiation (e.g., sorafenib or radiofrequency ablation) induces the selection of rare mutants and treatment-resistant sub-clones, culminating in the development of different genetic backgrounds at different times (temporal heterogeneity) [10,11]. As such, a particular tissue biopsy only mirrors a single spot of the tumor and is unlikely to represent the whole spectrum of somatic tumoral mutations due to the high degree of spatial and temporal heterogeneities. Furthermore, there are several problems associated with performing a liver biopsy, including the risk of spreading the tumor along the needle tract. In fact, biopsy in HCC is mostly recommended when radiological diagnosis is not conclusive and suspicion is high, but not for diagnostic or prognostic purposes.

**Figure 1:**
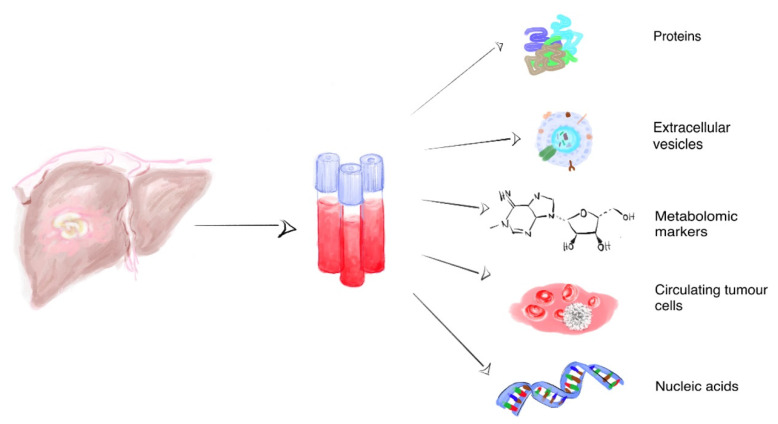
Liquid biopsy in HCC. Various forms of liquid biopsy, aiming at different tumor tracers, have been investigated in HCC. Some are still not commercially available or approved for HCC, or are still under experimental research, including cell-free DNA and cell-free RNA (cfDNA and cfRNA, respectively), circulating tumor cells (CTCs), extracellular vesicles (EVs), and metabolomics.

Altogether, the concept of liquid biopsy was developed to address the need for reliable, minimally invasive methods of diagnosis, prognosis and overall disease monitoring. It is a modality where body fluids samples, instead of solid tissue, are used for pathophysiological or molecular analyses. It has been introduced for many clinically relevant fields, including cancer research and, in general, any body fluids can be used as potential samples for liquid biopsy [12]. The term liquid biopsy can apply to cancer by-products including circulating tumor cells (CTC), cell-free DNA (cfDNA), cell-free RNA (cfRNA), microRNA (miRNA), extracellular vesicles (EVs), and tumor-derived metabolites (Figure 1). Others have reviewed the general role of liquid biopsies in HCC [13,14,15]. Nonetheless, the liquid biopsy research is dynamic and rapidly evolving, with many new data becoming available every year. The purpose of this article is to provide a comprehensive overview of the latest available data on the diagnosis, prognosis and predictive role of liquid biopsy in HCC. Finally, we will interpret available results as discussed in the text and as summarized briefly in Tables 1–3 after its associated text part.

## 2. Liquid Biopsy Biomarkers in HCC

### 2.1. Proteins

Tumor protein biomarkers include cytokines and proteins produced by both tumor cells and non-tumoral cellular components. The most commonly used and studied HCC biomarker is AFP. In brief, AFP has a similar molecular structure to albumin and contains one glycoprotein consisting of 591 amino acids. The gene for AFP is localized in chromosome 4 and its expression is elevated in the fetal period of human development as well as in adults with liver disease, including HCC [16]. According to its reactivity to lens culinaris agglutinin (LCA), AFP can be classified as AFP-L1, AFP-L2 and AFP L-3. AFP-L1 is mostly found in patients with chronic hepatitis and liver cirrhosis, while AFP-L3 is more commonly found in HCC patients [17,18,19]. Still, many recent studies have challenged the utility of AFP as an HCC biomarker, arguing for its rather low sensitivity and specificity [18,19,20,21,22,23]. Further, many HCC patients exhibit normal AFP serum levels, particularly during early stage disease [19,22]. As such, while AFP is sometimes still used in clinical practice as a risk assessment tool in cirrhotic patients with a high risk to develop HCC, clinical practice guidelines do not recommend AFP (or any other biomarker for that matter) for the diagnosis of HCC [24].

Des-gamma-carboxy-prothrombin (DCP) represents another potential protein-based circulating biomarker in HCC. In a phase 2 multicenter case-controlled study, early-stage HCC patients were shown to exhibit significant increases in total AFP, AFP-L3 and DCP. In particular, DCP was reported to showcase higher sensitivity and specificity in distinguishing HCC from other chronic liver diseases, including cirrhosis, when compared to AFP [18,22]. In terms of tumor volume, Nakamura et al. reported a better DCP-based predictive outcome, compared to AFP, in a case of HCC characterized by a massive tumor mass [25]. Interestingly, DCP performance is also superior to the one of AFP for HCC tumors with a diameter under 5 cm indicating a possible limitations since its reliability appears to greatly depend on tumor stage [25]. Parallel to Nakamura et al., Toyoda et al. compared AFP, AFP-L3 and DCP regarding HCC prognosis and reported that AFP-L3 and DCP likely mirror different features of HCC progression, prediction of patient outcome, and treatment efficacy [26]. HCC patients positive for all three markers were associated with the shortest survival and *vice versa*, patients were negative for AFP, AFP-L3 and DCP associated with increased survival [26].

Other circulating protein biomarkers studied in HCC patients include glypican-3 (GPC3), osteopontin, and vascular endothelial growth factor (VEGF). As a heparan sulfate proteoglycan, GPC3 interacts with different growth factors and participates in cell differentiation and migration, including of HCC tumor cells. GPC3 expression has been reported not to depend on HCC tumor size, suggesting a potential role as an early stage diagnostic HCC biomarker [27,28]. In addition, plasma levels of GPC3 have been suggested to predict HCC tumor recurrence [29]. Shimizu et al. also illustrated the utility of GPC3 as a biomarker and as a potential HCC vaccine immunotherapy target [30]. The combination of GPC3 with additional biomarkers, namely AFP, appears to further increase its diagnostic sensitivity and specificity [27,31]. Unfortunately, not all HCC cells express GPC3, while increased GPC3 expression is also found in squamous cell- and lung-carcinomas, clear cell tumors of the ovary, testicular germ cell tumors and melanomas [27,32,33]. In addition, significantly increased serum GPC3 levels have also been reported in patients with non-malignant liver diseases [19].

Neovascularization and angiogenesis are essential for tumor growth. Typically, tumors cannot grow over 2 mm in diameter without any neovascularization support. Angiogenic proteins, including members of the vascular endothelial growth factor (VEGF) family, like VEGF-A, play a vital role in tumor angiogenesis and tumor neovascularization. In fact, VEGF expression is found increased in HCC liver tissue. Of note, increased serum levels of soluble VEGF-A were associated with advanced HCC and shorter overall survival as discussed briefly by Zhou et al. [19]. Interestingly, liver VEGF was capable to indicate the presence of cancer with high accuracy, whereas AFP could identify the type of cancer as HCC, accordingly to Mukozu T. et al. [34].

In combination with AFP, the low specificity of VEGF in the diagnosis of advanced HCC has been shown to increase from 60% to 85%. The triple combination of AFP/VEGF and α-L-fucosidase (AFU) was associated in this pre-clinical study with a sensitivity of 100% and a specificity of 95% [35]. Of note this promising result is lacking so far the needed power, and it could be interesting that it is validated in larger patient cohorts. Interestingly, cancer cells appear not to represent the largest reservoir for VEGF, but rather the skeletal muscle [36]. As such, the combination of AFP and VEGF is prone to the same diagnostic drawbacks reported for AFP alone. VEGF was able to predict the overall survival in the SHARP study as an independent biomarker besides other, where 602 patients with advanced HCC receiving either oral sorafenib 400 mg twice a day or placebo were enrolled [37]. VEGF’s role as a biomarker for HCC lethality should be acknowledged based on additional studies like the SHARP study [37] and as recently published by Joo et al., underlining VEGF as a guide in future treatment decision-making [38].

Finally, glycoprotein osteopontin (OPN) represents another potential experimental HCC biomarker. Osteopontin, ubiquitously expressed in bone and epithelial cells, is also highly expressed by tumor cells, including in HCC. In this regard, Shang et al. reported that OPN alone was associated with good sensitivity and specificity values, hence the combination of AFP and OPN did in fact slightly improve the predictive sensitivity specificity to 95% and of 96%, respectively. The authors further demonstrated that OPN has high sensitivity in AFP-negative HCC [39].

Among the circulating protein biomarkers, AFP and AFP-L3, besides DCP and AFP in combination with OPN, represent widely used biomarkers in clinical use, despite some known limitations. A. Mohammed F.H. and L.R. Roberts discussed the drawbacks of AFP and why the AASLD is not in favor of endorsing AFP as a standalone biomarker in their guidelines, partly based on still missing evidence supporting its high sensitivity and specificity as an effective surveillance and diagnostic tool for HCC [40].

### 2.2. Metabolomic Markers

The development of “omics” technologies has made possible the study of hundreds of putative biomarkers simultaneously. Metabolomics is considered to be a powerful high-throughput platform to measure low molecular weight metabolites in biological samples (e.g., blood, urine, bile, ascites, tissue etc.). Thus far, it has already contributed to elucidate biochemical pathways playing a role in multiple human cancers, also offering a unique opportunity to discover novel biomarkers in this field [41,42]. Several analytical techniques can be employed, including nuclear magnetic resonance (NMR) [43], gas chromatography-mass spectrometry (GC-MS) [44], liquid chromatography-mass spectrometry (LC-MS) [45], capillary electrophoresis-mass spectrometry (CE-MS) [46], or various combinations of these analytical techniques [47].

In recent years, metabolomics has provided a large list of potentially novel biomarkers for HCC. A comprehensive analysis of all these biomarkers is beyond the purpose of this review and has already been compiled by others [48]. Overall, among the several metabolomic studies performed in HCC, few have assessed the diagnostic performances of each specific biomarker. Still, it appears that metabolomic biomarkers perform statistically better comparing with AFP in discriminating HCC from cirrhosis or HCC from healthy volunteers. In one of the first studies, a diagnostic panel of 18 metabolites was found to be able to differentiate HCC patients from healthy volunteers with an area under the curve (AUC) of 0.9275 [44]. In particular, the best model for discriminating between HCC and healthy controls was based on serum levels of 1-methyladenosine in combination with AFP, with an AUC of 0.95 [49]. Another model included endocannabinoids anandamide and palmitylethanolamide, with an AUC of 0.94 [50]. When discriminating between HCC and cirrhosis, a panel composed of formate, phytosphingosine and 3a, 6a, 7a, 12a-tetrahydroxy-5b-cholan-24-oic acid, exhibited an AUC of 1.000 and 0.995 for the identification and validation set, respectively [47]. Another model based on acetylcarnitine C3 and betaine was shown to exhibit an AUC of 0.98 in discriminating between HCC and cirrhosis [45]. Finally, a validation-designed study using a serum biomarker panel composed of tryptophan, arginine, glycine, and 2-hydroxybutiric acid yielded an AUC of 0.976 in discriminating between small HCC from pre-cancer cirrhosis [46].

Overall, while many metabolites appear to display excellent diagnostic performances in HCC, reproducibility stands as a major issue to address. The same holds true for the reporting of compensated or decompensated cirrhosis. In addition, validation cohorts have been used in a very small number of studies, most of them retrospective on nature and with no statistical adjustments for important confounding variables such as smoking status, alcohol consumption, lifestyle habits, physical activity, body mass index or waist circumference. Even so, a multi-centric, prospective study, using an NMR metabolomic approach, showed that when adjustments for these cofounders was performed, the AUC for discriminating HCC from healthy controls was only slightly better than AFP (75% vs. 73%) [43]. Nonetheless, differences in lifestyle habits, as well as environmental factors, appear to have a huge impact on the utility of metabolomic results and should be carefully balanced in future studies.

Similarly, in order to accelerate translation of metabolomic biomarkers into the clinics, future research should focus on pseudo-targeted and targeted approaches rather than the more commonly performed untargeted metabolomics experiments. As an example, in a urinary pseudo-targeted metabolomic approach, butyrylcarnitine and hydantoin-5-propionic acid yielded an AUC of 0.786 and 0.773 for the diagnosis of HCC in the discovery and validation cohorts, respectively [51]. In addition, in a single targeted metabolomic study, serum acetylcarnitine showed good potential in discriminating HCC patients from both healthy individuals (AUC of 0.803) and liver cirrhosis patients (AUC of 0.808) [52].

An ideal biomarker should also be able to discriminate between HCC and other types of liver cancer such as intrahepatic cholangiocarcinoma (CCA). However, the real challenge lies in correctly assessing combined hepatocellular-cholangiocarcinoma (cHCC-CCA) as cholangiolocarcinoma (CLC) and as intermediate cell carcinoma, potentially sharing features of both cell types [53]. However, a recent transcriptomic and metabolomic study found that a panel of 14 compounds could discriminate between intrahepatic cholangiocarcinoma and HCC with an accuracy of 78.13% [54], a performance value close to those of currently available imaging techniques. Recently, a urinary metabolic signature was proposed to associate with CCA. This signature was capable of distinguishing CCA among other cancer entities including hepatobiliary cancers [55]. It was also recently shown that in the serum, specific metabolites might also embody novel biomarkers to distinguish intrahepatic CCA from HCC, with an AUC of 0.89, 75% sensitivity, and 90% specificity, underlining the power of metabolomic biomarkers in biliary cancer *per se* [56].

### 2.3. Circulating Tumor Cells and Extracellular Vesicles

The use of circulating tumor cells (CTCs) for the diagnosis of cancer was approved in 2004 by the U.S. Food and Drug Administration (FDA). Since this method heavily relies on the availability of tumor-disconnected tumor cells in the peripheral blood circulation [57], FDA approval was restricted for the diagnosis of certain epithelial tumor entities, such as prostate, breast and colorectal cancer and limited to malign/metastatic tumor entities or cancer subtypes. Given the highly vascularized liver environment, the use of CTCs as biomarkers for HCC has been under scrutiny. For instance, two different meta-analyses have shown that CTCs associate with a poor prognosis and poor clinicopathologic parameters in HCC [58,59]. Epithelial-mesenchymal-mixed CTCs seem to play an important role in epithelial-mesenchymal transition in HCC, mixed CTCs appearing to play a vital factor for intrahepatic metastasis, and mesenchymal CTCs having the potential to predict extrahepatic metastasis [60]. In addition, both the positive rate and number of CTCs have been significantly correlated with tumor size and TNM staging in HCC patients [61]. Interestingly, CTC-based HCC surveillance could benefit from its combination with other techniques such as high-throughput microfluidic CTC-iChip—RNA-based digital PCR, to detect CTC-derived signatures. Kalinich et al. demonstrated such approach, where CTCs were first isolated via CTC-iCHip technology followed by RNA-based dPCR profiling yielding several targets that pointed towards the presence of HCC. This study reached promising predictive values of 83% (negative) and 86% (positive) in patients with underlying cirrhosis (no sensitivity and specificity was provided) [62]. It has also been suggested that AFP mRNA-positive CTCs may represent a novel predictor for HCC metastasis before and after hepatectomy [63]. Recently, single-cell mRNAseq in HCC patients yielded a genome-wide transcriptome profiling to confidently detect CTCs, while highlighting its potential role to monitor HCC heterogeneity and its ability to detect HCC driver genes [64]. These studies underline the potential synergistic effects of mRNA screening and CTC detection in HCC diagnosis and monitoring and hold potential for further exploitation.

A drawback of using CTCs as diagnostic biomarkers is their apparent lack of specificity, as its detection relies on the use of pan-cancer markers such as epithelial cell adhesion molecule (EpCAM) and creatine kinase (CK), among others [57]. Furthermore, only a small proportion (20%–35%) of HCCs express EpCAM [65], although at least a weak EpCAM expression in >10% of tumors was observed in 87 of 131 different tumor categories [66]. In this regard, the use of CTCs for monitoring anti-tumor therapy response and tumor recurrence is, perhaps, more promising. Shen et al. showed that elevated EpCAM^+^ CTCs correlated with poor survival of patients with unresectable HCC tumor after transcatheter arterial chemoembolization (TACE) [67]. In addition, detection of EpCAM^+^ CTCs in bloodstream prior to surgery predicts an elevated risk of HCC recurrence and shorter recurrence free survival after curative resection [68]. Similarly, following radical hepatic resection, elevated EpCAM^+^ CTCs and Treg/CD4^+^ cell levels were found to associate with a worst prognosis [69].

A somewhat newer concept, still in earlier experimental phases, suggests taking benefit of CTC recovery, followed by CTC expansion ex vivo. After sufficient expansion/proliferation, anti-tumoral drugs, as approved by the FDA, might be screened under standardized conditions to rule out unsuitable drugs, namely those with persisting resistance in patient’s derived CTCs [70,71]. This basic approach could (a) contribute to reduce public healthcare costs by avoiding the use of inefficient and expensive anti-tumoral drugs (b) lead to the identification of the most effective personalized anti-cancer drug and (c) decrease the amount of time until effective treatment for the benefit of the patient. Unfortunately, the major limiting factor for the use of these methodologies lies on how to induce a timely CTC expansion. In this regard, Khoo et al. recently published an experimental protocol aiming to reduce the time needed to expand CTCs, although most protocols still require months [71]. Of note, and until now, CTCs have not been recognised as a possible diagnostic tool for HCC by either AASLD or EASL.

When firstly identified, extracellular vesicles (EVs) were regarded as cellular waste or debris, without any biological function [72]. The current understanding is that EVs are a new cellular dimension of the so-called horizontal cell-cell communication, traveling to proximal and distal spaces with the ability of altering intracellular pathways with pathophysiological consequences [73,74,75,76]. For instance, tumor cells may use EVs to its advantage by enforcing tumor-induced immune suppression via the release of EVs [77]. Given the nature of EVs, particularly their nano sizes, a number of detection and analysis methods have been reported, with little standardization across studies. As a consequence, the International Society for Extracellular Vesicles (ISEV) released their first guidelines regarding experimental work on EVs in 2014 [78]. It was the first step for the growing EV community to standardize guidelines for EV characterization, addressing purity, desired EV isolation methods, and quantification procedures. The latest ISEV guideline MISEV2018 argues against the common use of the term exosomes and microvesicles (MVs)/microparticles (MPs) in favour of small EVs and large EVs, towards a more simplified nomenclature [79]. According to the ISEV guidelines, two major release mechanisms distinguish these two types of vesicles, namely EV shedding (large EVs) and the bulk release via exocytosis of multi-vesicular bodies (small EVs). Briefly, small EVs typically range in sizes between 30 nm and 100 nm in diameter, while large EVs measure above 100 nm, up to 1000 nm. Oncosomes, a third subtype of EVs, may eventually surpass them in size (up to 10 μm in diameter). Some EV markers have been postulated to be able to distinguish between small and large EVs, including CD63, HSP70, CD9, CD81 and integrins [80,81]. Nonetheless, it should be noted that the border of distinction between these EV populations is not sharp but rather fluent [78]. EVs possess a typical density of 1.13–1.19 g/mL [78], allowing for its separation and enrichment via sequential ultracentrifugation, among other methods [82]. EVs might be phenotypically characterized by their surface membrane proteins or their vesicular content, namely mRNA, lncRNA or proteins, by means of different commercially available kits [78].

Concerning the role of EVs as biomarkers in HCC, it was recently shown that surface marker HepPar1-positive EVs are found increased in HCC patients [83]. In addition, Julich-Haertel et al. showed that AnnexinV, EpCAM, ASGPR1 and CD133 simultaneously positive EVs could be detected in HCC and CCA, being further able to differentiate these two types of liver tumors from other cancer types, as well as patients with cirrhosis and healthy individuals, with a sensitivity of 81% and positive predictive value (PPV) of 73% (cirrhosis vs. HCC) [84]. In parallel, other studies have been focusing on EV content. For instance, it was recently shown that exosome-associated galectin-3-binding protein (LG3BP) provides higher AUC values for the diagnosis of HCC when comparing to AFP [85]. Another noteworthy publication showed a similar diagnostic value of EV hsa-miR-144-3p/hsa-miR-21-5p, found elevated in HCC (AUC of 0.78), compared to AFP (AUC of 0.626) [86]. Similarly, in a highly reproducible rodent HCC model, a panel of 4 miRNAs, namely miRNA-10b, miRNA-21, miRNA-122 and miRNA-200a, exhibited a higher AUC value in diagnosing HCC, comparing to AFP (0.943 vs. 0.826) [87]. In addition, although miR-148a (AUC of 0.860) alone was significantly superior to AFP (AUC of 0.665), the combination of miR-122, miR-148a, and AFP exhibited a higher diagnostic power in discriminating early HCC from liver cirrhosis in humans, with an AUC of 0.947 [88]. Additional and larger patient studies should ideally now be performed in order to fully establish the potential of EVs as reliable and reproducible biomarkers for HCC. Selected and associated publications are systematically summarized in Table 1.

### 2.4. Circulating Nucleic Acids in HCC

Cell-free nucleic acids are generally divided into “cell-free DNA” (cfDNA) and “cell-free RNA” (cfRNA), two broader terms that also account for circulating tumor DNA (ctDNA) and circulating tumor RNA (ctRNA). Both cfDNA and cfRNA are released into the blood stream through different mechanisms, including active secretion or through means of the apoptosis, necrosis, and lysis of CTCs. Once released into circulation, cfDNA forms short nucleosome-associated fragments or long fragments encapsulated within EVs. Given the relative instability of RNA, cfRNA is usually detected in association with proteins, proteolipid complexes and EVs [89].

**Figure 2:**
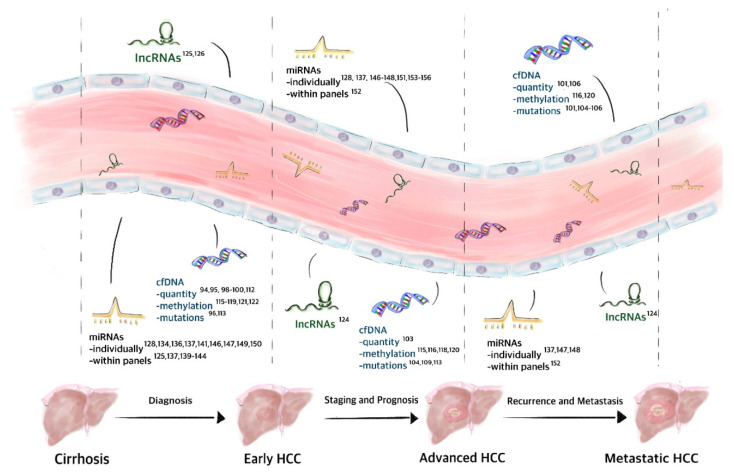
Role of circulating nucleic acids as biomarkers in HCC. Different types of circulating nucleic acids have already been described as potential biomarkers for the diagnosis and staging of HCC, as well as predictors of progression, recurrence and metastasis, among others, like treatment response. These circulating nucleic acids include lncRNAs; miRNAs used alone or in combinatory panels with other miRNAs or other molecules; as well as the detection of the quantity, hyper/hypomethylation status and different mutations in cfDNA.

The detection and analysis of circulating nucleic acids in blood may aid not only in disease diagnosis but also in guiding molecular targeted therapy, monitoring response to such or other treatments, as well as identifying mutations associated with drug resistance. Furthermore, unlike a tumor biopsy, circulating nucleic acids may provide useful information regarding tumor heterogeneity. The recent approval of the first companion diagnostic test based on the ctDNA content of a liquid biopsy for lung cancer by the FDA constituted a landmark in this field and is paving the way for the further exploitation of circulating nucleic acids as novel and minimally invasive disease biomarkers [90]. This approach is being evaluated through the quantification of cfDNA but more particularly through the detection of point mutations, indels, aberrant DNA methylation, and chromosomal aberrations, as well as cfRNA profiling and quantification [91] (Figure 2).

### 2.5. cfDNA

In 1948, Mandel and Metais first reported the detection of cfDNA in the serum of healthy individuals [92]. It then took approximately 50 years until the tumor biomarker potential of cfDNA started to emerge, with the identification of *KRAS* mutations in ctDNA isolated from patients with colorectal and pancreatic cancer [93]. Since then, many studies have been conducted to find the utility of circulating nucleic acids as liquid biopsies, including in the diagnosis of HCC. Earlier studies have focused on establishing the levels of cfDNA as a biomarker for detecting and staging HCC. For instance, in 2006, Iizuka et al. described that cfDNA levels were elevated in patients with hepatitis C virus (HCV)-associated HCC, and its diagnostic power was superior to that of serum AFP [94]. More recently, and because cfDNA levels can originate from multiple types of cancer, arguing against the specificity of single quantitation, the combination with additional markers has been reported, which appears to increase specificity. For instance, two mathematical models including cfDNA and AFP levels had a higher discriminative power in diagnosing HCC than that of a model with cfDNA or AFP alone [95,96]. This is particularly relevant considering that the predictive value of AFP alone (>20 ng/mL) has a sensitivity of ~60%, which is low [97].

Interestingly, a previous study revealed that the size profile of cfDNA from patients with HCC is shorter than that of non-tumor DNA [98]. Furthermore, this profile reverses to less fragmented cfDNA after HCC tumor resection [99]. It is noteworthy that cfDNA fragmentation also occurs in other types of cancer. With respect to this, a study found evidence that hepatic cfDNA showcases ends at specific genomic coordinates, reinforcing the notion that cfDNA fragmentation is a nonrandom process. Furthermore, quantitative assessment of cfDNA molecules has been shown to correlate with the relative abundance of tumor- or liver-derived DNA in plasma [100]. Nonetheless, and for the time being, the analysis of cfDNA methylation and the mutational landscape appears to offer more specificity.

An exome sequencing study showed that 83% of the mutations identified in the liver were also detected in cfDNA, which highlights the usefulness of cfDNA in the diagnosis of HCC [101]. In this regard, ultra-deep targeted sequencing for all exons of the 58 most frequently mutated genes in HCC reported an overall detection rate for tissue mutations in plasma cfDNA of 43% (9/21) [102]. A more recent study showed that all the mutations detected in plasma cfDNA matched HCC tissue DNA but not the opposite, pointing towards a high specificity but low sensitivity of plasma cfDNA as an HCC biomarker [103].

The ability to detect cfDNA mutations in patients with HCC also relates to clinical features of the disease. An earlier study showed that mutations typically detected in the hepatic tissue of patients with HCC were also detected in cfDNA (27% of the patients showed the presence of cfDNA and almost all patients (6/7) had either large tumor (≥5 cm diameter) or metastatic disease) [104]. Targeted deep sequencing, in turn, identified tumor-associated mutations for HCC in plasma cfDNA associating with vascular invasion [105]. Interestingly, cfDNA levels and the identification of 28 mutation variants correlated with the presence of metastases and survival in a cohort of 13 patients with advanced HCC [106].

Finally, the level of cfDNA as a liquid biopsy for HCC should ideally also arise from its ability to address intratumoral heterogeneity. This question was recently addressed through whole-exome sequencing and targeted deep sequencing in 32 multi-regional tumor samples and cfDNAs from five patients with HCC. The results showed that cfDNA was not as efficient as tissue samples in revealing disease heterogeneity [107]. Further studies using a large cohort and/or late-stage patients should be conducted to help shed more light into this issue.

*TP53* mutations are the most commonly found mutations in HCC tissue and cfDNA, with more than 120 non-unique alterations [108,109]. Using digital droplet PCR targeting, the TP53 c.747G > T (p.R249S) mutation, as well as CTNNB1 c.121A > G (p.T41A), CTNNB1 c.133T > C (p.S45A), and TERT c.-124C > T mutations, were identified in at least one mutation in 56.3% (27/48) of the cfDNAs obtained from patients with HCC. None of the aforementioned mutations were detected in the non-tumoral HCC tissue or in peripheral blood mononuclear cells (PBMCs) [110]. In particular, the *TP53* mutation at codon 249 appears to be highly specific. A recent study, making use of an experimental blood test, analyzed both genetic alterations and protein biomarkers in different types of stage I cancer. Their results showed that the TP53 c.747G > T (p.R249S) mutation was found in ~20% of the HCC plasma samples and in only ~3–4% of the pancreatic and stomach cancer samples, and it was not detected in a large cohort of 812 healthy controls. Mutations in *KRAS* and *CTNNB1* were also concomitantly found in HCC tissue and plasma samples [111]. Moreover, mutations in ERBB2 occurred more frequently in Hepatitis B (HBV)-positive patients [109]. A similar test, combining the analysis of cfDNA mutations with protein markers, was shown to be particularly useful in detecting early stage HCC in a high-risk asymptomatic population who were seropositive for the surface antigen of hepatitis B virus (HBsAg). In the training cohort, the HCC screen assay exhibited a sensitivity of 85% and specificity of 93% in diagnosing HCC. In the validation cohort, the HCC screen assay exhibited 100% sensitivity and 94% specificity [112].

The human telomerase reverse transcriptase (TERT) gene encodes for the catalytic subunit of telomerase that will act together with multiple molecular partners to maintain telomere homeostasis and, as such, chromosomal integrity. Highly recurrent mutations have been found in in the promoter of *TERT*, providing a mechanism for *TERT* reactivation and oncogenic transformation. Of note, *TERT* promoter mutations is one of the most common genetic alterations in HCC, also found in cfDNA of patients with HCC, with similar prevalence. Male patients with HCV and/or alcohol-induced cirrhosis were identified as the patient group with the highest prevalence of *TERT* promoter mutations, either within the tumor or in the cfDNA [113]. As such, querying for *TERT* promoter mutations in cfDNA may allow for the early detection of HCC in populations at risk of HCC.

Since tumor suppressor genes are methylated early during tumorigenesis, analysis of DNA methylation patterns may embody key diagnostic value. With respect to this, and similar to the cfDNA quantification and mutational landscape analysis, cfDNA aberrant methylation allows for diagnostic and prognostic insights in HCC, and might reveal information regarding the tumor size and risk of metastasis or recurrence [114]. A recent study identified a DNA methylation marker panel enriched in HCC tissues, which was replicated in cfDNA of the same patients. A diagnostic prediction model with 10 selected methylation markers showed high specificity (>83%) and sensitivity (>90%) correlating with tumor burden, treatment response, and disease stage. The model further differentiated patients with HCC from those with hepatitis B virus (HBV)/HCV infection and fatty liver disease [115]. Suppressor of cytokine signaling 3 (*SOCS3*) methylation in both HCC tissue and plasma was also found to be associated with tumor size and differentiation, metastasis, and recurrence, as well as with poorer prognosis [116].

Analysis of DNA methylation patterns could also be used to screen populations at risk of HCC. In a recent study, RAS association domain family protein 1A (*RASSF1A*) methylation was largely detected in the cfDNA of patients with HCC (52.04%), but not as much in patients with liver cirrhosis or chronic hepatitis B or in healthy controls (13.33%, 4.44%, and 3.75%, respectively). Of note, the *RASSF1A* methylation exhibited better sensitivity and specificity (52.0% and 91.5%, respectively) in distinguishing HCC from chronic hepatitis B compared with AFP (48.0% and 73.9%, respectively) [117]. Interestingly, *RASSF1A* has been combined with three additional candidates (APC, COX2, and miR-203) in a methylation status prediction model (AUC of 0.87) with a sensitivity and specificity of 84.2% and 83%, respectively, in the diagnosis of HBV-related HCC, which is, again, superior to the currently used AFP detection model [118]. Likewise, a 32-gene diagnostic model, detecting genome-wide 5-hydroxymethylcytosines, accurately distinguished patients with early HCC or small tumours (e.g., ≤2.0 cm) from non-HCC subjects (AUC of 0.884), including high risk patients with liver cirrhosis or chronic hepatitis B (AUC of 0.846) [119].

Hypomethylation of cfDNA may also serve as a biomarker of HCC. In particular, several studies have reported increased levels of hypomethylated-type transposase domain containing long interspersed nuclear element 1 (LINE-1) in cfDNA of patients with HCC. This is associated with clinical pathologic features such as HBsAg positivity, tumor size, or AFP levels. LINE-1 hypomethylation is also associated with poor survival of patients with HCC and with early recurrence and poor prognosis upon curative resection when combined with the measurement of *RASSF1A* hypermethylation [120].

Alterations in cfDNA methylation, associated with the initiation and progression of HCC, have been described for many other genes, including *p15*, *p16*, *APC*, *SPINT2*, *SFRP1*, *p16INK4a*, *TFPI2*, *GSTP1*, *SEPT9*, *VIM*, and *FBLN1,* as well as in CpGs sites [14,121,122,123]. Altogether, these results support the hypothesis that analysis of ctDNA methylation status embodies novel putative biomarker tests for HCC.

The use of ctDNA as a liquid biopsy still poses several limitations, particularly in the early detection of cancer, where ctDNA levels are low. However, the lack of standardized protocols for preanalytical sample preparation and ctDNA purification and analysis is the major limitation in the diagnosis of HCC. While large prospective, well-controlled studies on HCC cfDNA profiling are highly warranted, the promising results obtained so far testify to the potential of cfDNA as a superior tool for tumor diagnosis and monitoring. Selected and associated publications are systematically summarized in Table 2.

### 2.6. cfRNA

Most known classes of RNA have been detected in circulation, with long noncoding RNAs (lncRNAs) and microRNAs (miRNAs) being the most studied as putative disease biomarkers. LncRNAs are typically >200 nucleotides in length and play a key role in epigenetic regulation. Due to their ability to form stable secondary structures, lncRNAs can be found in body fluids and have been studied as potential biomarkers for different diseases, including HCC. The long intergenic nonprotein coding RNA 974 (Linc00974) has been found increased in the serum of patients with HCC [124], whereas lncRNA–CTBP exhibited high sensitivity and specificity in differentiating HCC from both healthy controls and patients with cirrhosis [125]. In a recent study, three circulating lncRNAs, namely LINC00152, RP11-160H22.5, and XLOC014172, combined with AFP, were able to distinguish HCC from chronic hepatitis patients or healthy controls with an AUC of 0.986 and 0.985, respectively [126].

### 2.7. miRNA

miRNAs are a class of short, single-stranded, noncoding RNAs (18–24 nt) that play a key role in the posttranscriptional regulation of gene expression. By doing so, miRNAs modulate many pathophysiological processes, including cancer. They can be found in circulation enclosed in EVs or associated with proteins (typically argonaute 2 (AGO2) and nucleomorphin 1 (NPM1)) or lipoproteins (e.g., HDL). Many circulating miRNAs, including *miR-106b*, *miR-21*, *miR-200a*, *miR-122*, *miR-223*, *let-7f*, and *miR-155* have already been described to associate with HCC, with promising diagnostic and prognostic value [127,128]. Overall, more than 70 miRNAs have already been suggested as candidate biomarkers for HCC [129]. Of note, special attention has been paid to *miR-122* and *miR-192* because of their liver specificity and enrichment. In fact, both these markers account for over 70% of the total miRNA pool in the liver [130]. While this could mean that *miR-122* and *miR-192* would lack specificity, as any type of liver disease will affect their release into circulation, it is becoming apparent that partitioning of the release of *miR-122*, that is, its incorporation into EVs or association with carrying proteins, might be specific to the type of liver tissue damage [131,132,133]. Future studies should elucidate whether the combination of data addressing the partitioning of *miR-122* with its expression might improve its relevance as a biomarker for HCC.

A meta-analysis based on 24 articles revealed that the expression levels of *miR-21*, *miR-122*, and *miR-199* appear to rank among the most selective for the diagnosis of HCC [134]. However, it should be noted that conflicting results on circulating *miR-21* have been published, with some suggesting upregulation [135,136] and others suggesting downregulation in HCC [110,137]. Nonetheless, serum *miR-21* levels might have a better predictive role as a putative biomarker for HCC when used in conjunction with additional biomarkers. In fact, similar to cfDNA, the combination of different miRNAs into a biomarker panel, or its combination with select clinical parameters, appears to increase sensitivity for HCC diagnosis. This is the case, for instance, when combining *miR-21* with *miR-26a*, *miR-27a*, *miR-122*, *miR-192*, *miR-223* and *miR-801*, which altogether exhibit a high diagnostic accuracy for HCC. Furthermore, this miRNA panel was able to differentiate between patients with HBV and cirrhosis and healthy subjects, with an AUC of 0.84, 0.88, and 0.94, respectively [138,139]. Other examples of combined miRNA panels include serum levels of *miR-375*, *miR-10a*, *miR-122*, and *miR-423*, found elevated in patients with HCC comparing with healthy controls (AUROC of 0.995) [140]; *miR-101-3p*, *miR-1246*, and *miR-106b-3p*, which exhibited high diagnostic accuracy for HCC when patients with cirrhosis (AUC of 0.99) were compared with control subjects (AUC of 1.00) [141]; or *miR-122*, *miR-142-3p* and *miR-486* in distinguishing HCC from cirrhosis in patients with chronic Hepatitis C (AUC = 0.94) [142]. Interestingly, a recent study combining eight miRNAs showed a sensitivity of 97.7% and a specificity of 94.7% in discriminating the presence of HCC in high risk patients, allowing the detection of 98% stage I HCC cases [143].

Of note, *miR-26a* and *miR-101*, combined with AFP, exhibited a better sensitivity for HCC than AFP alone [110]. Similarly, the combination of *miR-92-3p*, *miR-107*, and *miR-3126-5p* with AFP was highly effective in differentiating patients with early stage HCC (AUC of 0.988) and patients with a low level of AFP in HCC (AUC of 0.989) from healthy individuals, even more so than the three miRNAs panel alone [144]. Last but not least, a panel of *miR-122*, *miR-885-5p*, and *miR-29b*, associated with AFP testing, was shown to exhibit high diagnostic accuracy for the early detection of HCC in a normal population, whereas combining *miR-122*, *miR-885-5p*, *miR-221*, and *miR-22* with AFP allowed for the accurate diagnosis of early HCC in cirrhosis [139]. A recent meta-analysis evaluating the significance of circulating miRNAs in the diagnosis of HCC indeed suggests that the combination of miRNAs with AFP elicits superior diagnostic performance [145].

Circulating levels of select miRNAs also correlate with different clinical features and tumor properties. For instance, increased plasma levels of *miR-21* and *miR-224* in patients with HCC was found to correlate with clinical stage and distant metastasis and larger tumor size and recurrence, respectively [146,147]. Moreover, serum *miR-1246* was found increased in HCC patients with early tumor recurrence within 12 months after hepatic resection, exhibiting high potential as a recurrence and prognostic biomarker of HCC [148]. Indeed, several studies have revealed that cfRNA levels reflect tumor dynamics and burden, with important implications for disease monitoring. For instance, both *miR-224* and *miR-500* are highly expressed and detected in the plasma of patients with HCC, but its circulating levels significantly decreased following resection [146,149]. On the contrary, *miR-148a* is downregulated and therefore detected in low quantities in the plasma of patients with HCC compared with patients with cirrhosis and healthy controls, and tumor resection upregulated the expression of *miR-148a* [150].

Recent studies have focused on the role of circulating miRNAs in HCC as predictors of clinical outcomes after treatment, thus helping in finding a personalized therapy for individual patients. A recent study showed that serum levels of *miR-106b*, *miR-107*, and *miR-133b* were elevated in patients with HCC who responded to TACE, with *miR-26a* being elevated in nonresponders. *miR-26a* and *miR-133b* exhibited the highest diagnostic performance, with *miR-133b* further distinguishing complete responders from partial responders and non-responders (AUC ≥ 0.90) [151]. Other studies have indicated that a combinatory analysis of plasma levels of *miR-21*, *miR-26a*, and *miR-29a-3p*, or even *miR-122* alone, may predict early TACE refractoriness in patients with TACE-treated HCC [152]. It was also suggested that high expression levels of *miR-122*, as well as low expression levels of *miR-26a* and *miR-29a*, could constitute a poor prognostic marker for patients with HCC undergoing radiofrequency ablation [153,154]. With respect to chemotherapeutic drugs, circulating *miR-221* levels have been reported to associate with sorafenib treatment response in patients with HCC [155], whereas recent results from the RESORCE (NCT01774344) clinical trial, identified several miRNAs, namely *miR-30a*, *miR-22*, *miR-125b*, *miR-200a*, *miR-374b*, *miR-15b*, *miR-107*, *miR-320*, and *miR-645*, associated with increased overall survival of patients with HCC who are treated with regorafenib [156]. This highlights its possible usefulness in identifying patients most likely to respond to treatment. Nonetheless, and similar to cfDNA, the most important limitation to the implementation of cfRNAs in the clinical setting still pertains to the pre-analytical and analytical steps. Selected and associated publications are systematically summarized in Table 3.

## 3. Further Directions

Given the high incidence of HCC, the search for non-invasive biomarkers is crucial in modern diagnostics of this disease. Such markers should not only be useful in very early diagnosis of the disease but also in prognosis and monitoring of its course and treatment. Based on a review of the literature, it appears that such features are present within a liquid biopsy; however, this is not a flawless method. First off, methodological difficulties in biomarker determination loom large for scientists. The currently used methods require special preparation of biological material and highly specialized laboratory equipment. For this reason, there is a need to intensify efforts to develop a fast, simple, and inexpensive analytical technique. In light of the experimental and clinical studies available, the lack of such a method is a serious limitation on the use of liquid biopsy in clinical practice. For this reason, we have formed the opinion that future research should focus on this topic and this is supported by the fact that many groups of researchers have demonstrated the high specificity and sensitivity of circulating markers, but their introduction for use in the clinic is highly unlikely until a simple analytical method has been developed.

## 4. Conclusions

As discussed in this review, there are some good preclinical arguments for the urgent need of liquid biopsy in HCC diagnosis, screening and surveillance. Some of the reviewed techniques will likely help physicians to perform a more accurate diagnosis through synergistic effects base on novel liquid biopsy based methods and of previously established techniques such as ultrasound, CT, and MRI. The image-based analysis provides the operator with visible information of a lesion. However, it requires an experienced professional to interpret the seeable lesion. However, subjectivity could potentially be an issue. Additional tests based on liquid biopsy, as discussed, might led to a synergy to overcome image-based drawbacks. Ideally, in liquid biopsy, only the cut-off value should determine the diagnosis and increase image based diagnosis confidence.

Recently, the term “liquid biopsy” has evolved to include a variety of newly developed methods used for HCC diagnosis. As discussed above, some of the techniques show good preliminary results, whereas others appear promising but have not yet reached conclusive results. Additional biomarkers, capable of being detected by mass spectrometry or RNA arrays will probably be established soon. Altogether, screening for various types of nucleotides, such as cfRNA and miRNA, could soon be incorporated into different clinical practice guideline, including those of EASL and AASLD. In addition, a conceptual new direction has recently emerged, namely EVs. Only a few studies have been conducted so far promoting the idea to use EVs as a novel liquid biopsy marker for HCC, but the field is promising.

Overall, we cannot predict the technique that will finally hit the market and satisfy the demand for non-invasive HCC biomarkers in the form of a liquid biopsy. It could be very well that the combination of EV technology and screening of nucleotides within EVs, as discussed for EV RNA, could be a game-changer. Of note, reliably working liquid biopsies will have many advantages for the patient’s welfare and will likely be associated with lower healthcare costs.

## Figures and Tables

**Table 1:** HCC: hepatocellular carcinoma, AUC: area under the curve; AFP: alpha-fetoprotein; DCP: Des-gamma-carboxy-prothrombin; * clinical AFP cut-off; ** 40 mAU/mL cut-off DCP; CHB: chronic hepatitis B; CTC: circulating tumor cells; EVs: extracellular vesicles; OPN: osteopontin; ^#^ depicted ROC provided, but no number given; ^§^ pooled sensitivity and pooled specificity; ° was not able to differentiate between HCC and CCA; ″ values not reported in the studies but estimated from AUC curves; Ref.: references.

Type of Biomarker	Sample	Number of Patients (HCC)	Control (Number)	Sensitivity and Specificity (AUC)	Study Design	Ref.
AFP-L3	Serum	836 (419)	Compensated cirrhosis controls (417)	42%; 97% (0.72) *	Phase 2 biomarker case-controlled	[18]
AFP	Serum	836 (419)	Compensated cirrhosis controls (417)	59%; 90% (0.83) *	Phase 2 biomarker case-controlled	[18]
DCP and AFP	Serum	836 (419)	Compensated cirrhosis controls (417)	86%; 63% (0.XX) *	Phase 2 biomarker case-controlled	[18]
DCP and AFP	Serum	207 (55)	G1: normal liver biochemistry (48) G2: confirmed non-cirrhotic chronic hepatitis (51) G3: proven cirrhosis and compensated liver disease (53)	90%; 95% (0.92)	Retrospective	[22]
AFP	Serum	1709 (1361)	Hepatitis C common etiologic factor (348)	62%; 97% (0.93) *	Retrospective	[25]
DCP	Serum	1709 (1361)	Hepatitis C common etiologic factor (348)	58%; 97% (0.81) **	Retrospective	[25]
DCP and AFP	Serum	1709 (1361)	Hepatitis C common etiologic factor (348)	82%; 91% (0.81) *, **	Retrospective	[25]
VEGF	Serum	124 (59)	Hepatitis C virus related liver cirrhosis (28)	98%; 46% (XX) ^#^	Retrospective	[34]
VEGF, AFP and AFU	Serum	124 (59)	Hepatitis C virus related liver cirrhosis (28)	100%; 95% (XX) ^#^	Retrospective	[34]
OPN	Plasma	312 (131)	Cirrhosis/CHB (96)	82%; 96% (XX)	Retrospective	[39]
OPN and AFP	Plasma	312 (131)	Cirrhosis/CHB (96)	95%; 96% (XX)	Retrospective	[39]
16 metabolites	Serum	336 (114)	Healthy individuals (222)	75.4%; 80.6% (XX)	Prospective	[43]
18 metabolites and AFP	Urine	40 (20)	Healthy male cases (20)	90%; 85% (0.92) ″	Retrospective	[44]
acetylcarnitine C3 and betaine	Tissue	50 (50)	Adjacent noncancerous (50) Distal noncancerous (50)	97%; 84% (0.98) ″	Retrospective	[45]
tryptophan, arginine, glycine, and 2-hydroxybutiric acid	Serum	106 (50)	Liver cirrhosis (26) Healthy individuals (31)	98%; 97% (0.99)	Retrospective	[46]
formate, phytosphingosine and 3a,6a,7a,12a tetrahydroxy-5b-cholan-24-oic acid	Serum	103 (43)	Liver cirrhosis (42) Healthy individuals (18)	100%; 100% (1.00)	Retrospective	[47]
1-methyladenosine and AFP	Plasma	79 (41)	Healthy individuals (38)	92%; 88% (0.95) ″	Retrospective	[49]
endocannabinoids anandamide and palmitylethanolamide	Serum	128 (69)	Healthy individuals (31) Cirrhosis (28)	84%; 90% (0.94)	Retrospective	[50]
butyrylcarnitine and hydantoin-5-propionic acid	Urine	54 (33)	Liver cirrhosis (21)	91%; 52% (0.77)	Retrospective	[51]
acetylcarnitine	Serum	58 (18)	Liver cirrhosis (20) Healthy individuals (22)	74%; 79% (0.80)	Prospective	[52]
glycine, aspartic acid, SM (42:3) and SM (43:2)	Serum	40 (20)	Cholangiocarcinoma (20)	75%; 90% (0.89)	Retrospective	[56]
CTC	Peripheral Blood	2256 (998)	Healthy and various hepatic and tumorous diseases (1258)	67%; 98% (XX) ^§^	Meta-analysis of total 20 studies	[59]
large EVs	Serum	214 (86)	Cirrhosis (49)	81%; 47% ° (0.73)	Retrospective	[84]
small EVs	Serum	57 (24)	CHB (16)	90%; 80% (0.78) ″	Retrospective	[86]
small EVs and AFP	Serum	180 (50)	Cirrhosis (40)	86%; 88% (0.93)	Retrospective	[88]

**Table 2:** HCC: hepatocellular carcinoma, AUC: area under the curve, cfDNA: “cell-free DNA”, HCV: hepatitis C virus, AFP: alpha-fetoprotein, HBV Hepatitis B, HBsAg: surface antigen of hepatitis B virus, US: ultrasonography, *RASSF1A*: RAS association domain family protein 1A, *SEPT9*, Septin 9 gene.

Type of DNA Biomarker	Sample	Number of Patients (HCC)	Control (Number)	Sensitivity and Specificity (AUC)	Study Design	Ref.
cfDNA quantity	Serum	98 (52)	HCV carriers without HCC (30) and HCV-negative non-cancer patients (16)	69.2%; 93.3% (0.90)	Retrospective	[94]
cfDNA quantity + AFP	Plasma	86 (24)	HBV-related liver fibrosis patients (62)	87%; 100% (0.98)	Retrospective	[95]
cfDNA size	Plasma	225 (90)	healthy individuals (32)	80%; 94% (0.93)	Retrospective	[98]
cfDNA size	Plasma	106 (53)	healthy individuals (22)	43.4%; 100% (0.705)	Retrospective	[99]
cfDNA size + AFP	Plasma	106 (53)	healthy individuals (22)	79.2%; 100%	Retrospective	[99]
cfDNA mutations	Plasma	43 (37)	healthy individuals (6)	65%; 100% (0.92)	Retrospective	[96]
cfDNA mutations	Plasma	8 (8)	matched HCC tissue DNA (8)	30%; 100%	Retrospective	[103]
cfDNA mutations + AFP (+)	Plasma	43 (37)	healthy individuals (6)	53%; 100% (0.86)	Retrospective	[96]
cfDNA mutations + AFP (−)	Plasma	43 (37)	healthy individuals (6)	73%; 100% (0.96)	Retrospective	[96]
cfDNA mutations + protein markers	Plasma	135 (65)	non-HCC cases in HBsAg-positive patients with AFP/US positive results (70)	85%; 93% (0.928)	Retrospective	[112]
cfDNA mutations + protein markers	Plasma	331	Prospective study	100%; 94%	Prospective	[112]
cfDNA methylation	Plasma	1275 (715)	healthy individuals (560)	85.7%; 94.3% (0.966)	Retrospective	[115]
cfDNA methylation	Plasma	658 (383)	healthy individuals (275)	83.3%; 90.5% (0.944)	Retrospective	[115]
cfDNA methylation (*RASSF1A*)	Serum	188 (98)	chronic hepatitis B patients (90)	52%; 91.5% (0.718)	Retrospective	[117]
cfDNA methylation	Plasma	1120 (335)	non-HCC cases (785)	89.6%; 78.9% (0.923)	Retrospective	[119]
cfDNA methylation	Plasma	1194 (220)	non-HCC cases (385)	82.7%; 76.4% (0.884)	Retrospective	[119]
cfDNA methylation (*SEPT9*)	Plasma	186 (51)	cirrhotic patients without HCC (135)	98%; 64.4%	Retrospective	[122]
cfDNA methylation (*SEPT9*)	Plasma	103 (47)	cirrhotic patients without HCC (56)	93.6%; 75%	Retrospective	[122]
cfDNA methylation + AFP	Serum	188 (98)	chronic hepatitis B patients (90)	83.7%; 78.9% (0.852)	Retrospective	[117]
cfDNA methylation + miRNA	Plasma	226 (123)	chronic hepatitis B with and without cirrhosis (53) and healthy individuals (50)	84.2%; 83% (0.87)	Retrospective	[118]

**Table 3:** HCC: hepatocellular carcinoma, AUC: area under the curve, lncRNA–CTBP: long non-coding RNA-C terminal binding protein, androgen responsive, LAMP2: lysosomal-associated membrane protein 2, ETR: early tumour recurrence, TACE: transcatheter arterial chemoembolization, AFP: alpha-fetoprotein.

Type of RNA Biomarker	Sample	Number of Patients (HCC)	Control (Number)	Sensitivity and Specificity (AUC)	Study Design	Ref.
lncRNA–CTBP + miR-16-2 + miR-21-5p + LAMP2	Serum	158 (78)	healthy individuals (44) and chronic hepatitis B patients (36)	79.5%; 100% (0.938)	Retrospective	[125]
miR-106b	Serum	335	non-HCC cases (310)	90%; 66.7% (0.855)	Retrospective	[128]
miR 21-5p	Serum	40 (23)	chronic hepatitis patients (17)	100%; 81.2% (0.943)	Retrospective	[136]
miR 21-5p	Serum	453 (175)	healthy individuals (136) and chronic hepatitis B/liver cirrhosis patients (142)	82.1%; 83.9% (0.849)	Retrospective	[147]
miR-199-a	Serum	40 (23)	chronic hepatitis patients (17)	54.5%; 100% (0.856)	Retrospective	[136]
miR-224	Plasma	142 (87)	healthy individuals (55)	93.1%; 80% (0.908)	Retrospective	[146]
miR-1246	Serum	209 (121)	HCC without ETR (31)	54.1%; 77.4% (0.762)	Retrospective	[148]
miR-148a	Plasma	346 (155)	healthy individuals (95)	97.9%; 92.7% (0.98)	Retrospective	[150]
miR-148a	Plasma	346 (155)	liver cirrhosis patients (96)	89.6%; 89% (0.919)	Retrospective	[150]
miR-133b	Serum	51 (51)	Patients with HCC (partial responders (17) and non-responders (18) to TACE)	93.8%; 88.2% (0.919) and 100%; 94.4% (0.997)	Prospective	[151]
miR-21, miR-26a and miR-101 + AFP	Serum	137 (52)	healthy individuals (43)	87%; 78% (0.914)	Retrospective	[137]
miR-26a and miR-101 + AFP	Serum	137 (52)	chronic hepatitis patients (42)	72.5%; 86.7% (0.854)	Retrospective	[137]
miR-101-3p + miR-106b-3p and miR-1246	Plasma	128 (62)	healthy individuals (25)	100%; 100% (1)	Retrospective	[141]
miR-101-3p + miR-106b-3p and miR-1246	Plasma	128 (62)	liver cirrhosis patients (41)	100%; 92.9% (0.99)	Retrospective	[141]
miR-122, miR-142-3p and miR-486	Serum	60 (20)	liver cirrhosis patients (20)	80%; 95% (0.94)	Retrospective	[142]
Panel of 8 miRNAs	Serum	1517 (345)	healthy individuals (1033) and chronic hepatitis/liver cirrhosis patients (139)	97.7%; 94.7% (0.99)	Retrospective	[143]
